# Physician Health: Results and Caveats from Surveys in Austria, Switzerland and Germany

**DOI:** 10.1192/j.eurpsy.2022.89

**Published:** 2022-09-01

**Authors:** F. Wurst, H.-J. Rumpf, N. Thon, P. Beschoner

**Affiliations:** 1 Psychiatric University Hospital Basel, Basel, Switzerland, Psychiatry Department, Basel, Switzerland; 2 Ulm University Medical Center, Department Of Psychosomatic Medicine And Psychotherapy, Ulm, Germany

**Keywords:** physician health, alcohol use, prevalence

## Abstract

Background: Surveys assessing alcohol use among physicians most commonly employed the Alcohol Use Disorders Identification Test (AUDIT) or the AUDIT-C. As with other screeners, prevalence estimation is dependent on the accuracy of the test as well as choice of the cut-off value. The aim of the current study is to use samples from various countries derive more precise prevalence estimates of alcohol problems in physicians by correcting for false positive and false negative results using samples from various countries Method: At the Congress of the German Association of Psychiatry, Psychotherapy and Psychosomatics, 2005 in Berlin, 1800 questionnaires, which included the AUDIT-C were distributed among the attending participants. 936 questionnaires (52%) were returned. Also, the data are compared to a second study, performed in Salzburg, Austria to further elucidate the situation. The screening results will be presented and compared to the values when using a correction- formula using data from a general population sample on sensitivity and specificity of the AUDIT-C. Results: Based on the results of AUDIT-C and using a cut-off of 5 for both sexes, 24.1% of the sample of 887 physicians of the German sample are problematic drinkers (14.7% in female and 32 % in male physicians). Using a correction formula leads to markedly lower rates: 6.1% (all), 3.7% (female), 8.1% (male). Discussion: In this large sample, findings clearly confirm that uncorrected screening results lead to severe over-estimation of the prevalence of problematic drinking in physicians. The corrected prevalence rates are lower than in the general population.

**Disclosure:**

No significant relationships.

